# Methods and consequences of including reduction in greenhouse gas emission in beef cattle multiple-trait selection

**DOI:** 10.1186/s12711-019-0459-5

**Published:** 2019-04-29

**Authors:** Stephen A. Barwick, Anthony L. Henzell, Robert M. Herd, Bradley J. Walmsley, Paul F. Arthur

**Affiliations:** 1 Animal Genetics and Breeding Unit (AGBU), University of New England, Armidale, NSW 2351 Australia; 2 NSW Department of Primary Industries, Livestock Industries Centre, Armidale, 2351 Australia; 3 NSW Department of Primary Industries, Elizabeth Macarthur Agricultural Institute, Menangle, 2568 Australia

## Abstract

**Background:**

Societal pressures exist to reduce greenhouse gas (GHG) emissions from farm animals, especially in beef cattle. Both total GHG and GHG emissions per unit of product decrease as productivity increases. Limitations of previous studies on GHG emissions are that they generally describe feed intake inadequately, assess the consequences of selection on particular traits only, or examine consequences for only part of the production chain. Here, we examine GHG emissions for the whole production chain, with the estimated cost of carbon included as an extra cost on traits in the breeding objective of the production system.

**Methods:**

We examined an example beef production system where economic merit was measured from weaning to slaughter. The estimated cost of the carbon dioxide equivalent (CO_2_-e) associated with feed intake change is included in the economic values calculated for the breeding objective traits and comes in addition to the cost of the feed associated with trait change. GHG emission effects on the production system are accumulated over the breeding objective traits, and the reduction in GHG emissions is evaluated, for different carbon prices, both for the individual animal and the production system.

**Results:**

Multiple-trait selection in beef cattle can reduce total GHG and GHG emissions per unit of product while increasing economic performance if the cost of feed in the breeding objective is high. When carbon price was $10, $20, $30 and $40/ton CO_2_-e, selection decreased total GHG emissions by 1.1, 1.6, 2.1 and 2.6% per generation, respectively. When the cost of feed for the breeding objective was low, selection reduced total GHG emissions only if carbon price was high (~ $80/ton CO_2_-e). Ignoring the costs of GHG emissions when feed cost was low substantially increased emissions (e.g. 4.4% per generation or ~ 8.8% in 10 years).

**Conclusions:**

The ability to reduce GHG emissions in beef cattle depends on the cost of feed in the breeding objective of the production system. Multiple-trait selection will reduce emissions, while improving economic performance, if the cost of feed in the breeding objective is high. If it is low, greater growth will be favoured, leading to an increase in GHG emissions that may be undesirable.

**Electronic supplementary material:**

The online version of this article (10.1186/s12711-019-0459-5) contains supplementary material, which is available to authorized users.

## Background

Concern about global warming has focussed attention on reducing greenhouse gas (GHG) emissions from farm animals [1, 2], particularly in beef cattle [3, 4]. An important aim of animal breeding is to improve the economic productivity of animals. In the absence of improvements in feed efficiency, productivity gains are often associated with increases in feed intake, which are associated with increased overall GHG emissions [5] and GHG emissions per unit of product [6].

Many studies have shown that a reduction in GHG emissions can be achieved via breeding [6–8]. Wall et al. [7] examined strategies that can be applied in UK livestock. Quinton et al. [6] reported that decreases in total GHG emissions and GHG emissions per unit of product accompanied increases in productivity in beef cattle. Many of the GHG emission studies described in the literature have limitations, i.e. they use breeding objectives that inadequately specify feed intake, assess the consequences of breeding for a particular trait only, or examine the effect of productivity improvement on emissions for only part of the production chain.

In this article, we present multiple-trait selection strategies for reducing GHG emissions in beef cattle for the whole production chain from weaning to slaughter. We focus on reducing GHG emissions that are associated with feed intake, since criteria for reducing emissions independently of feed intake (referred to as residual GHG emissions) are still under development. We evaluate the outcomes and consequences of reducing emissions at the level of both the individual animal and the production system, by incorporating different levels of carbon price in the breeding objective. We also examine the consequences at different levels of feed cost, since this was shown earlier to be a major factor affecting the ranking of beef cattle for economic merit [9].

## Methods

### Definitions

Animal breeding is principally concerned with improving breeding value for net merit, which is usually expressed in economic terms. Commonly, the selection criterion is an index of estimated breeding values (EBV) $\widehat{{\mathbf{u}}}$ that are available for an industry [10, 11]. The genetic variance–covariance matrix for deriving the index has partitions ${\mathbf{G}}_{11}$ for variances and covariances among EBV in the index, ${\mathbf{G}}_{22}$ for variances and covariances among traits in the breeding objective, and ${\mathbf{G}}_{12} \left( { = {\mathbf{G}}_{21}^{\prime } } \right)$ for covariances between the EBV criteria and the traits of the objective [12]. Values for the breeding objective traits for animal i are estimated as $\widehat{{\mathbf{g}}}_{{\mathbf{i}}} = {\mathbf{G}}_{{{\mathbf{21}}}} {\mathbf{G}}_{{{\mathbf{11}}}}^{{ - {\mathbf{1}}}} \widehat{{\mathbf{u}}}_{{\mathbf{i}}}$ [9]. When the index is linear, weights for the EBV of the index are derived as ${\mathbf{b}} = {\mathbf{G}}_{11}^{ - 1} {\mathbf{G}}_{12} {\mathbf{v}}$, where **v** are economic values of the breeding objective traits. The index is commonly derived as ${\mathbf{b}}^{\prime } \widehat{{\mathbf{u}}}_{{\mathbf{i}}}$, but it can be equally derived as ${\mathbf{v}}^{\prime } \widehat{{\mathbf{g}}}_{{\mathbf{i}}}$.

### The commercial production system

The commercial production system that underlies the breeding objective in beef cattle can be pasture-based or grain-based, or it can include a mix of both systems post-weaning. In the example taken for this article, pasture-raised steers and surplus heifers are finished on grain for 100d and slaughtered at 22 months of age (Table 1). In grazing systems, there are two types of annual feed period—i.e. a period when the available feed is limited, meaning any increase in feed requirement will require additional feed to be supplied, and a period when additional feed is not needed because the available feed in that period is surplus to the animal’s needs. In the latter case, we consider that the cost of feed is zero since no additional feed is needed. In all other cases, the feed cost is considered non-zero.

**Table 1:** Key characteristics for the example self-replacing beef cattle production system producing 100-d feedlot finished steers

Characteristic	Value
Calendar	
Month of joining	Oct
Start month of limited feed period^a^	Jan
End month of limited feed period	Sept
Carbon price	
Price of carbon, $/ton CO_2_-e	20
Young animals	
Age at weaning, m	7
Growth rate at pasture in limited feed period (relative to surplus feed period), − 1.0 to 1.0	0.7
Cost of extra feed at pasture, $/ton	130
Quality of extra feed at pasture, MJ/kgDM	8
Period of feedlot finishing, d	100
Steer live weight at feedlot entry, kg	450
Cost of extra feedlot feed, $/ton	270
Quality of extra feedlot feed, MJ/kgDM	10
Age at finished sale, m	22
Steer live weight at finished sale, kg	640
Finished steer sale price, cents/kg carcass	456
Cows (averages)	
Weaning %	85
Weight loss from calving to joining, kg	50
Live weight at joining, kg	650
Mean condition score at joining^b^	3−
Min. acceptable condition score at joining^b^	2+
Max. acceptable condition score at joining^b^	4+
Cost of extra cow feed, $/ton	130
Quality of extra cow feed, MJ/kgDM	8
Live weight of surplus cows, kg	550
Sale price for surplus cows, $	990

From the example used by Barwick et al. [9], with carbon priced at $20/ton CO_2_ equivalent

^a^All of the year except when surplus feed is available

^b^Scored on a 15-point scale from 1 − (emaciated) to 5 + (obese)

### Breeding objective

**Fig. 1:**
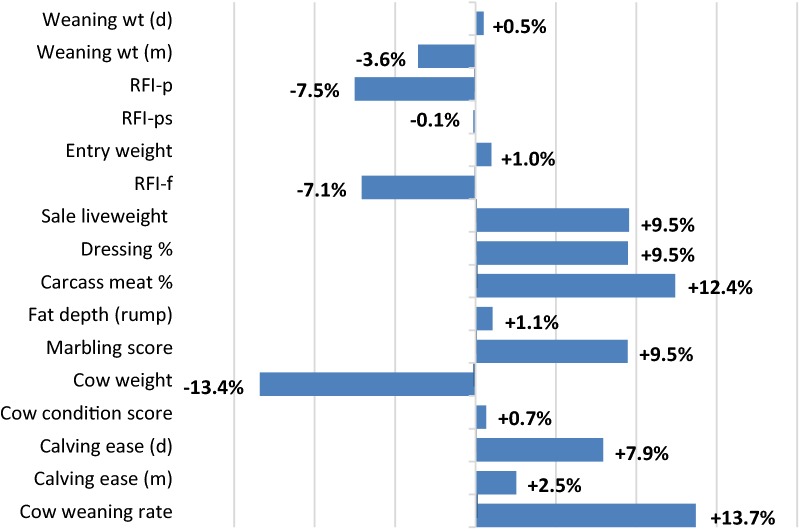
Economic importance of breeding objective traits for the example of beef cattle production system in Table 1^a–c^. (^a^Units are the value of a standard deviation of trait change relative to the value of a standard deviation of change in all breeding objective traits [9], ^b^Carbon priced at $20/ton CO_2_-e, ^c^GHG emissions are assumed associated with RFI traits)

The breeding objective examined here is net return per cow (i.e. returns net of all feed and management costs), which was assessed over the period from weaning to sale of the finished animal. Traits in the breeding objective are those that directly influence commercial production profitability. In this study, these are weaning weight (direct and maternal), residual feed intake (RFI) at pasture when pasture is limited, RFI at pasture when the feed available is surplus to the animal’s needs, feedlot entry liveweight, RFI in the feedlot, sale liveweight, dressing percentage, saleable meat percentage, fat depth (on the rump), marbling score, cow liveweight, cow condition score, calving ease (direct and maternal) and cow weaning rate. The economic importance of these traits is illustrated in Fig. 1 for the example beef cattle production system taken in our study. The economic importance of traits is calculated as:

$$ \left( {\text{v}_{i} \sigma_{{\text{G}i}} /\varSigma |\left. {\text{v}_{i} \sigma_{{\text{G}i}} } \right|} \right) \times 100, $$

where ${\text{v}}_{i}$ are the trait economic values and $\upsigma_{{\text{G}i}}$ are the genetic standard deviations for the *i*th trait of the breeding objective [12]. Economic importance for each trait is expressed as a percentage of the sum of the absolute values of the product for all traits [11]. Economic importance encompasses both the economic value of traits and the genetic variation that is available for each trait in the production system. The cost of the additional feed needed for a change in these traits is included in the economic values of the traits that influence feed requirement. Changes in feed requirement are assessed using the equation systems of Freer et al. (see Chapter 1 of [13]). Residual feed intake traits describe differences in feed intake that occur at the same weight and weight gain of animal.

**Fig. 2:**
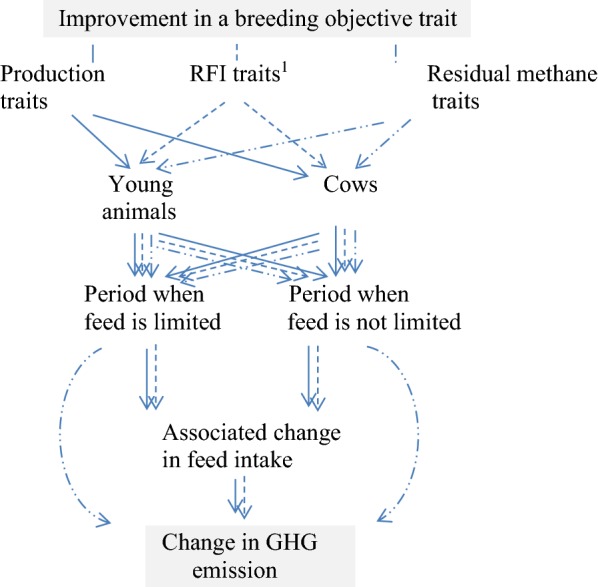
Pathways to a change in GHG emission from improvement in a breeding objective trait. Results are presented with and without an assumed association between GHG and residual feed intake (RFI) traits

The possible pathways to changes in emissions for a production system are in Fig. 2. Emissions commonly change when a trait in the breeding objective is improved, including when there is a change in either the residual GHG traits or the RFI traits. The cost of the increased carbon in the emissions is incorporated in the economic values of the breeding objective traits. Residual GHG traits are not considered in the described example because criteria for these are not yet available. Most reports suggest that GHG emissions decrease when RFI traits decrease [14–16]. However, Herd et al. [17] suggests that GHG emissions could increase when RFI decreases if, for example, the digestibility of the diet also increases. Because of the uncertainty surrounding this aspect, we present results with and without an assumed association between changes in RFI traits and GHG emission.

### Estimation of the cost of GHG emissions for breeding objective traits

When decreasing GHG emissions is part of the breeding objective, the cost of increasing emissions is included in the economic value determined for each breeding objective trait, according to the following steps:

- Estimation of the change in feed needed as a consequence of change in the breeding objective trait.
- Estimation of the change in methane emissions associated with the change in feed, and augmentation of this to account for the increase in non-methane GHG emissions for any period during which cattle are in feedlots.
- Conversion of the augmented change in methane to CO_2_-e.
- Application of the nominated price of carbon to the CO_2_-e change.

### Prediction of GHG emission levels from feed intake

Feed-associated GHG emissions are predicted from the feed intake of the production system. For animals at pasture, the phenotypic relationship of Charmley et al. [18] was used, i.e.:

$$ {\text{MP}} = 20.7\left( { \pm 0.28} \right) \times {\text{DMI,}} $$

where MP is the production of methane in g/day and DMI is the dry matter intake in kg/day. This suggests that 0.0207 kg of methane is associated with an increase in feed intake of 1 kg. Then, the recommended global warming constant for methane (28; i.e. a measure of how much heat methane traps in the atmosphere over a specific time horizon relative to carbon dioxide) (Intergovernmental Panel on Climate Change [4]) is used to determine that 0.5796 kg of CO_2_-e is associated with 1 kg of feed intake. Equation 7 of Johnson et al. [19] is used to predict GHG emissions when animals are in the feedlot, i.e.:

$$ {\text{CH}}_{4} = 9.90 - 1.54{\text{LOI}} - 0.02{\text{DE,}} $$

where ${\text{CH}}_{4}$ is % methane, expressed as a fraction of the animal’s diet gross energy intake, ${\text{LOI}}$ is the level of feed, expressed relative to the energy required for maintenance, and ${\text{DE}}$ is % diet digestible energy. Average daily ${\text{LOI}}$ of steers in the feedlot for the example of this article is equal to 2.32. The predicted % methane is used with feed intake (MJ/d) and the quality of feedlot feed (MJ/kg DM) to derive the amount of methane in kg generated in the feedlot. Emissions in the feedlot period are augmented by 11% to account for non-methane GHG emissions (e.g. nitrous oxide) that occur especially under feedlot conditions [4]. The augmented amount of methane is converted to CO_2_-e for assessing the cost of the CO_2_-e associated with each breeding objective trait.

### Residual GHG emission traits

Residual GHG emission traits are not included in the example described in this paper because criteria for these are not available and there is little knowledge on their association with other traits. Excluding residual GHG emission traits will not affect results if they are not correlated with other traits, at least until criteria for these traits are available. If the production system has no feedlot phase, residual GHG emissions in the feedlot are not part of the breeding objective.

### Price of feed

The price used for estimating the cost of feed in the breeding objective has a major impact on the ranking of beef cattle for economic merit [9]. This price can differ between young animals and cows, and for feed of different qualities (MJ/kg DM). If the production system is able to provide the additional feed needed for genetic improvement, the price of feed is a nominated $/ton for each quality of feed. If the production system is not able to provide the additional feed, the price of feed used is an implied price that reflects the way the production system would need to change to meet the increased feed requirement. In the case in which stocking rate would need to be reduced by reducing cow numbers, for example, the implied price is the production system’s net return per cow ($) divided by the feed intake per cow (MJ).

When reducing GHG emissions is part of the breeding objective, the cost of the CO_2_-e associated with feed intake change is an additional cost in the calculation of the economic values of the breeding objective traits. In the example of this article, the production system is assumed to be able to supply the additional feed needed, and the same price was used for estimating the costs of the additional feed needed when RFI traits change.

### Price of carbon

The cost of the carbon associated with change in the breeding objective traits is an additional cost to the production system. The carbon price (per ton CO_2_-e) used should take any consensus on this that is reached into account.

### Available estimated breeding values

The most relevant EBV to include in selection indexes are those that are routinely available to industry. In Australia, the EBV that are available to industry (listed here together with % accuracy levels that typically occur for active sires of the Angus breed) are: 200d weight in kg (91), 200d weight maternal in kg (79), 400d weight in kg (90), 600d weight in kg (90), net feed intake-post-weaning (NFI-p) in kg/d (62), net feed intake-feedlot (NFI-f) in kg/d (63), carcass eye muscle area in cm^2^ (78), carcass rump fat depth in mm (79), carcass retail beef % (74), carcass intra-muscular fat % (75), mature cow weight in kg (85), birth weight in kg (94), calving ease (direct) % (75), calving ease (maternal) % (62), days to calving in d (58), and scrotal size in cm (86) (see [10] for a complete description). The NFI-p and NFI-f EBV are net feed intake EBV for the residual feed intake traits RFI-p and RFI-f. Currently, EBV for residual GHG emission traits are not available.

### The genetic variance–covariance matrix

The genetic variance–covariance matrix for deriving selection indexes includes the partitions ${\mathbf{G}}_{11}$, ${\mathbf{G}}_{22}$ and ${\mathbf{G}}_{12}$ described above. These partitions may overlap. The matrix used for the example in this article relates to *Bos taurus* breeds and is similar to that used by Barwick et al. [9]. Details can be obtained from the authors on request.

### Changes in individual traits due to selection

Changes in individual traits due to selection are calculated with standard software. For the example in this article, the MTIndex software of J. van der Werf was used. The information that is assumed available for each EBV aligns with the above-mentioned accuracies of the EBV of active Angus sires of the Australian industry. In cases in which changes in individual traits need to be estimated and the accuracies of the EBV differ between individuals, the procedure described by Barwick et al. [9] is used.

### Changes in the production system due to selection

#### Total GHG emissions, total product, total feed and $ net return

Changes in the breeding objective traits that result from selection can change the feed needs of animals and hence the resulting CO_2_-e. The changes in total GHG emissions, total product, total feed and $ net return for the production system are estimated by summation over the breeding objective traits, as shown in Table 2. For young animals, these total sums can be for animals at pasture, in the feedlot, or overall. Similarly, the total for cows is summed over the breeding objective traits for cows. If increased feed is needed for cows to be in acceptable joining condition, this is also added. Further summing over young animals and cows gives the total sums for the production system (Table 3).

**Table 2:** Method of accumulating changes in traits for assessing the effects of selection on emissions from the beef cattle production system

Breeding objective traits (T_i_)	Change in trait (∆T)	Associated change in feed intake (MJ)			Associated change in GHG emission (g)^a^		
		Young animal pasture (∆PF^)b^	Young animal feedlot (∆FF)^c^	Cow pasture (∆CF)^d^	Young animal pasture (∆PG)	Young animal feedlot (∆FG)	Cow pasture (∆CG)
T_1_	∆T_1_	∆PF_1_	∆FF_1_	∆CF_1_	∆PG_1_	∆FG_1_	∆CG_1_
T_2_	∆T_2_	∆PF_2_	∆FF_2_	∆CF_2_	∆PG_2_	∆FG_2_	∆CG_2_
T_n_	∆T_n_	∆PF_n_	∆FF_n_	∆CF_n_	∆PG_n_	∆FG_n_	∆CG_n_
Total		Σ(∆PF_i_)	Σ(∆FF_i_)	Σ(∆CF_i_)	Σ(∆PG_i_)	Σ(∆FG_i_)	Σ(∆CG_i_)

^a^Emissions associated with feed intake over 12 months (in this paper, calculated with and without an association between RFI traits and GHG emission)

^b^Feed requirement and RFI change at pasture; restricted to the period of limited feed

^c^Feed requirement and RFI change over the feedlot period

^d^Feed requirement change over 12 months, including feed requirement for cows to be in joining condition; restricted to the period of limited feed

**Table 3:** Method of assessing effects of selection on GHG emissions from the beef cattle production system

Performance measure	Performance level^a^
After selection	
Young animal pasture feed (PF_A_), MJ	PF_B_ + Σ(∆PF_i_)
Young animal feedlot feed (FF_A_), MJ	FF_B_ + Σ(∆FF_i_)
Young animal total feed, MJ	PF_A_ + FF_A_
Cow pasture feed (CF_A_), MJ	CF_B_ + Σ(∆CF_i_)
Total feed (TF_A_), MJ	PF_A_ + FF_A_ + CF_A_
Young animal GHG emission at pasture (PG_A_), g	PG_B_ + Σ(∆PG_i_)
Young animal GHG emission in the feedlot (FG_A_), g	FG_B_ + Σ(∆FG_i_)
Young animal total GHG emission, g	PG_A_ + FG_A_
Cow GHG emission at pasture (CG_A_), g	CG_B_ + Σ(∆CG_i_)
Total GHG emission (TG_A_), g	PG_A_ + FG_A_ + CG_A_

Effects of selection	Before selection (X_B_)	After selection (X_A_)	% Change
Total product^b^, kg	KG_B_	KG_A_	(X_A_/X_B_ × 100) − 100
Total feed, MJ	TF_B_	TF_A_	
Total GHG emissions, g	TG_B_	TG_A_	
$ net return^c,d,e^	DNR_B_	DNR_B_ + Σ(VTC_i_)	
Product per unit of feed^e^, kg/MJ	KG_B_/TF_B_	KG_A_/TF_A_	
GHG per unit of product, g/kg	TG_B_/KG_B_	TG_A_/KG_A_	
GHG per unit of feed, g/MJ	TG_B_/TF_B_	TG_A_/TF_A_	
$ net return per unit of feed^e^, $/MJ	DNR_B_/TF_B_	[DNR_B_ + Σ(VTC_i_)]/TF_A_	
$ net return per unit of GHG^e^, $/g	DNR_B_/TG_B_	[DNR_B_ + Σ(VTC_i_)]/TG_A_	

^a^Includes level of performance of the production system before selection (per cow): young animal pasture feed (PF_B_), young animal feedlot feed (FF_B_), total young animal feed (PF_B_ + FF_B_), cow pasture feed (CF_B_), total feed (TF_B_), young animal GHG emission at pasture (PG_B_), young animal GHG emission in the feedlot (FG_B_), cow GHG emission (CG_B_), total GHG emission (TG_B_), total product (KG_B_), $ net return (DNR_B_)

^b^Expressed as equivalent steer beef, before (KG_B_) and after (KG_A_) selection

^c^Feed price before selection is based on a land value of $400/DSE (feed required by a 50 kg dry sheep (9.7 MJ/head/d) [20]), leased at 7% p.a. [21]

^d^Total value of trait change, Σ(VTC_i_) = Σ(∆T_i_ from selection x economic value of T_i_); ∆T_i_ assumes that selection intensity, i is equal to1

^e^Uses total feed, before and after selection, assessed over the limited feed period

#### GHG emissions per unit of product and per unit of feed

**Fig. 3:**
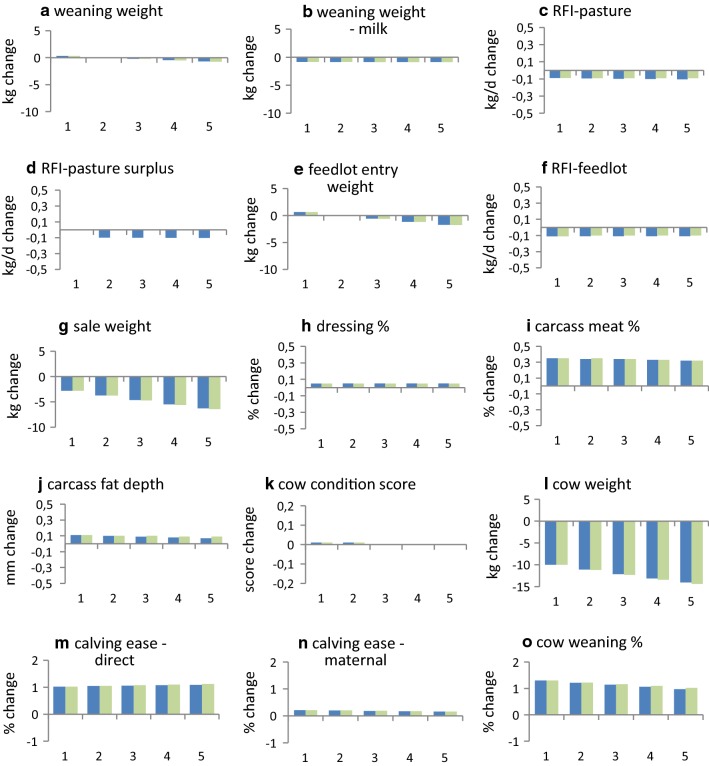
Effect of carbon price on individual trait responses to selection for the example beef cattle production system in Table 1^a,b,c,d^. (^a^For a single generation of selection with selection intensity *i* = 1, ^b^1 = $0/ton CO_2_-e; 2 = $10/ton CO_2_-e; 3 = $20/ton CO_2_-e; 4 = $30/ton CO_2_-e; 5 = $40/ton CO_2_-e, ^c^blue colour bars include GHG association with residual feed; green colour bars exclude GHG association with residual feed, ^d^Response in carcass marbling score was 0.02 of a score for all levels of carbon price)

Reductions in GHG emissions per unit of product and per unit of feed are achieved by reducing total GHG emissions or by increasing the productivity or feed intake of the production system. The change in percentage for each of these (Fig. 3) is based on the total sums before and after selection (Table 3).

#### Product per unit of feed, and $ net return per unit of product, feed or methane

Changes in product per unit of feed and in $ net return per unit of product, feed or methane measure the effects of selection on the biological and economic efficiency of the production system. The change in percentage for each of these is based on the total sums before and after selection (Table 3).

## Results

### Numerical example

Key characteristics for the example beef cattle production system analysed here, in which animals are sold for slaughter after 100d of feedlot finishing, are in Table 1. We chose this production system as an example because of the importance of pasture-grain systems to beef production globally, and because pasture-grain systems have not previously been described in the literature on breeding objectives. Tables 2 and 3 show how GHG emissions are accumulated across traits regardless of the production system. Tables 4 and 5 show how this occurs for the example in this paper. Calculations are in Additional file 1.

**Table 4:** Accumulated changes in traits for the example beef cattle production system in Table 1

Breeding objective traits	Change in traits	Associated feed intake, MJ^a^			Associated C0_2_-e, kg^b^		
		Young animal pasture	Young animal feedlot	Cow pasture	Young animal pasture	Young animal feedlot	Cow pasture
Weaning live weight-direct, kg	− 0.194	1.4	0	0.06	0.58	0	0.07
Weaning live weight-maternal, kg	− 0.866	0	0	− 70	0	0	− 5
RFI-pasture, kg/d	− 0.098	− 116	0	0	− 7	0	− 1.8
RFI-pasture surplus, kg/d	− 0.100	0	0	0	− 1.3	0	− 0.31
Entry live weight, kg	− 0.580	− 21	18	− 4	− 6	1.3	− 0.85
RFI-feedlot, kg/d	− 0.11	0	− 110	0	0	− 4	0
Sale live weight, kg	− 4.63	0	− 195	0	0	− 14	− 2
Dressing %	0.05	0	0	0	0	0	0
Carcass meat %	0.34	0	0	0	0	0	0
Carcass fat depth, mm	0.09	0	0	0	0	0	0
Carcass marbling score^c^	0.02	0	0	0	0	0	0
Cow live weight, kg	− 12.14	0	0	− 882	0	0	− 60
Cow condition score^d^	0	0	0	0	0	0	0
Calving ease-direct, %	1.06	0	0	0	0	0	0
Calving ease-maternal, %	0.18	0	0	0	0	0	0
Cow weaning %	1.14	267	232	87	33	17	0.64
Total		131	− 55	− 870	19	− 0.37	− 70

Characteristics of the production system are in Table 1

Methods for accumulating changes in traits are in Table 2

^a^Accumulated over the limited feed period

^b^Accumulated over 12 months; RFI traits are assumed to have associated GHG emissions

^c^Scored on a 12-point scale from 1 (least) to 12 (most) [22]

^d^Scored on a 15-point scale from 1- (emaciated) to 5 + (obese)

**Table 5:** Responses to selection for the example beef cattle production system in Table 1

Performance measure	Performance level
After selection	
Young animal pasture feed intake (PF_A_), MJ	12,740
Young animal feedlot feed intake (FF_A_), MJ	10,987
Young animal total feed intake, MJ	23,728
Cow pasture feed intake (CF_A_), MJ	31,328
Total feed intake (TF_A_), MJ	55,056
Young animal GHG emission at pasture (PG_A_), kg CO_2_-e	932
Young animal GHG emission in feedlot (FG_A_), kg CO_2_-e	33
Young animal total GHG emission, kg CO_2_-e	965
Cow GHG emission at pasture (CG_A_), kg CO_2_-e	2262
Total GHG emission (TG_A_), kg CO_2_ equiv.	3228

Effects of selection	Before selection (X_B_)	After selection (X_A_)	% Change
Total product, kg	172	171	− 0.64
Total feed intake, MJ	55,851	55,056	− 1.42
Total GHG emission, kg CO_2_-e	3279	3228	− 1.58
$Net return^a^	398	422	6.14
Product per unit of feed, kg/MJ	0.003097159	0.003121866	0.80
GHG per unit of product, kg CO_2_-e/kg	18.9	18.7	− 0.95
GHG per unit of feed, kg CO_2_-e/MJ	0.058726425	0.058631001	− 0.16
$Net return per unit of feed^a^, $/MJ	0.007126103	0.007672676	7.67
$Net return per unit of GHG^a^, $/kg CO_2_-e	0.12134407	0.130863803	7.85

Characteristics of the production system are in Table 1

Methods for assessing effects are in Table 2

All quantities are assessed over 12 months unless indicated

^a^Assessed using total feed, before and after selection, over the period of limited feed

#### Selection when the feed needed to improve breeding objective traits is expensive

**Fig. 4:**
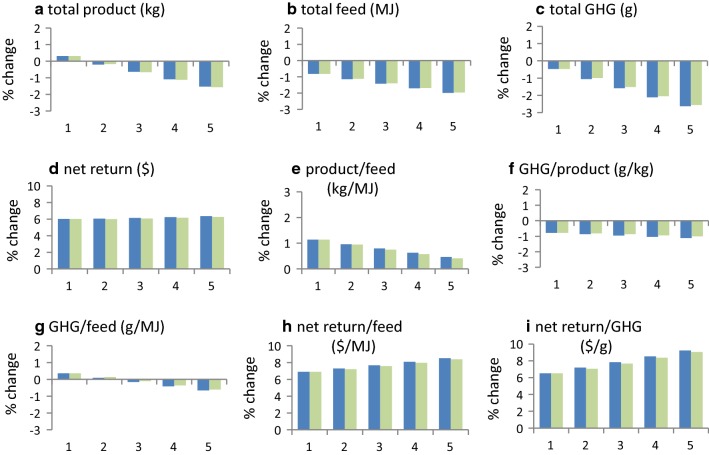
Effect of carbon price on production system responses to selection for the example beef cattle production system in Table 1^a,b,c^. (^a^For a single generation of selection with selection intensity *i* = 1, ^b^1 = $0/ton CO_2_-e; 2 = $10/ton CO_2_-e; 3 = $20/ton CO_2_-e; 4 = $30/ton CO_2_-e; 5 = 40/ton CO_2_-e, ^c^blue colour bars include GHG association with residual feed; green colour bars exclude GHG association with residual feed)

In production systems in which the cost of feed for the breeding objective is expensive (e.g. Table 1), selection is able to simultaneously reduce GHG emissions and increase economic performance. For the example in Table 1, total GHG emissions decreased by 1.1, 1.6, 2.1, and 2.6% per generation (relative to when carbon was not costed) when carbon in the breeding objective was priced at $10, $20, $30 and $40/ton CO_2_-e, respectively (Fig. 4c). Trends in responses for individual traits (Fig. 3), and for the production system (Fig. 4), did not differ markedly when no association was assumed between changes in RFI traits and GHG emissions. Emissions per unit of product and per unit of feed also decreased (Fig. 4f and g). When the costs of GHG emissions were not included (i.e. when carbon was priced at $0/ton CO_2_-e), reductions in total feed of the production system (0.8%) (Fig. 4b) and in total GHG emissions (0.5%) were small (Fig. 4c), and GHG emissions per unit of feed (0.4%) increased slightly (Fig. 4g). Selection was able to reduce total GHG emissions of the production system without appreciably affecting net returns for the production system (Fig. 4d). Selection was able to improve virtually all the traits, and most traits were not affected by carbon price. The traits that were most affected by carbon price were cow weight, which decreased by up to 14 kg as carbon price increased, and sale weight, which decreased by up to 6 kg (Fig. 3).

#### Selection when the feed needed to improve breeding objective traits is inexpensive

**Fig. 5:**
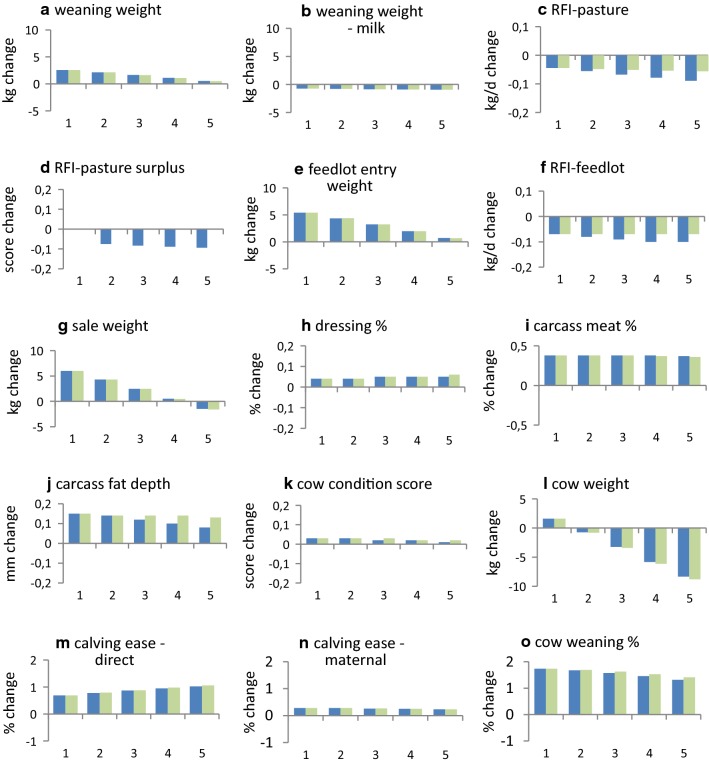
Effect of carbon price on individual trait responses to selection when feed price in the breeding objective is 30% lower than shown in Table 1^a,b,c,d^. (^a^For a single generation of selection with selection intensity *i* = 1, ^b^1 = $0/ton CO_2_-e; 2 = $20/ton CO_2_-e; 3 = $40/ton CO_2_-e; 4 = $60/ton CO_2_-e; 5 = $80/ton CO_2_-e, ^c^blue colour bars include GHG association with residual feed; green colour bars exclude GHG association with residual feed, ^d^Response in carcass marbling score was 0.02 of a score for all levels of carbon price)

**Fig. 6:**
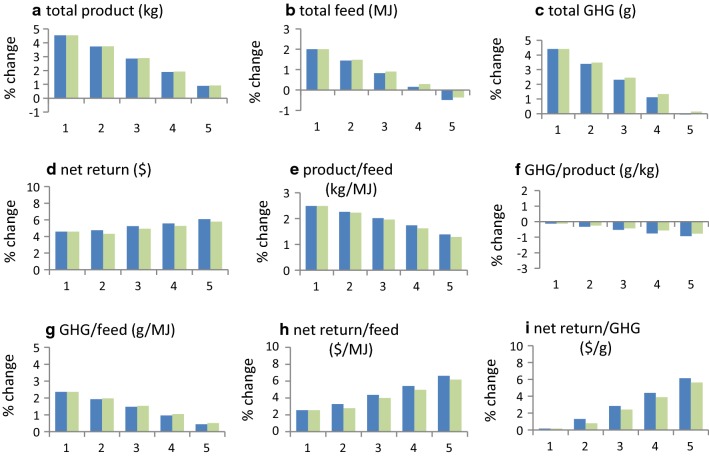
Effect of carbon price on production system responses to selection when feed price in the breeding objective is 30% lower than shown in Table 1^a,b,c,d^. (^a^For a single generation of selection with selection intensity *i* = 1, ^b^1 = $0/ton CO_2_-e; 2 = $20/ton CO_2_-e; 3 = $40/ton CO_2_-e; 4 = $60/ton CO_2_-e; 5 = $80/ton CO_2_-e, ^c^the blue colour bars include GHG association with residual feed; the green colour bars exclude GHG association with residual feed, ^d^$ net return is assessed using feed prices of Table 1)

In production systems in which the cost of feed for the breeding objective is inexpensive, such as when the price of feed was 30% lower than that in Table 1, selection was able to reduce total GHG emissions only if carbon price was equal to or higher than about $80/ton CO_2_-e (Fig. 6c). When the costs of GHG emissions were not included, total GHG emissions increased by 4.4% in one generation (Fig. 6c), or by ~ 8.8% in 10 years. GHG emissions per unit of product and per unit of feed both decreased as carbon price increased, although each remained greater than when feed was expensive (Figs. 5 and 6). Total GHG emissions increased and these increases were sizeable for all but the highest level of carbon price considered (Fig. 6c). Net returns to the production system increased as carbon price increased (Fig. 6d). Again, trends were not very different regardless of whether or not GHG emissions were assumed to be related to change in RFI traits (Figs. 5 and 6).

Figure 5a, d, f and j shows that weight traits increased less with increased carbon price, and selection effects were weaker on other performance traits. The increase in cow weight became negative at a carbon price of just less than $20/ton CO_2_-e (Fig. 5j). The increase in sale weight became negative at a carbon price between $60 and $80/ton CO_2_-e (Fig. 5f).

## Discussion

The price at which a production system can produce or purchase feed is the main factor that determines the cost of feed for the breeding objective. Our results show that the ability of selection to reduce GHG emissions depends on this. When feed for the breeding objective is at least moderately expensive, multiple-trait selection can reduce total GHG emissions and simultaneously increase economic performance (Figs. 3 and 4). The reduction in GHG emissions (Fig. 4c) is of the order of 3 to 5% in 10 years. Larger reductions are possible when reducing GHG emissions is the only objective of breeding [23], but this ignores that breeding usually aims at increasing economic performance.

When the cost of feed for the breeding objective is inexpensive, selection can only reduce GHG emissions if the carbon price used in the breeding objective is high. The carbon price at which GHG emissions decreased in the current example was about $80/ton CO_2_-e (Fig. 6c). The carbon price at which sale weight decreased was between $60 and $80/ton CO_2_-e (Fig. 5f). The resulting ~ 8.8% increase in GHG emissions over 10 years emphasises the importance of fully costing feed for selection decisions, and this is more critical when there is a need to also reduce GHG emissions. It shows that the management that is modelled for the development of breeding objectives needs to be close to optimal. Societal and political pressures to reduce GHG emissions may make production systems that underuse available feed, or which underestimate the cost of feed, unsustainable.

To date, the actual cost of feed for beef production has not been examined in the literature and needs to be estimated for different types of beef systems. Feed for intensive systems is often thought to be expensive, while that for grazing systems is considered less expensive. Table 6 lists several possible relationships between some characteristics of the commercial production system and whether the feed needed to improve breeding objective traits is expensive or inexpensive. Use of this type of classification, together with better information on the costs of feed, could assist selection in beef cattle.

**Table 6:** Possible relationships between the cost of feed needed to improve breeding objective traits and characteristics of the beef cattle production system under grazing

Feed cost for trait improvement	Characteristics of the commercial production system
Expensive	
Available feed fully used e.g.	High stocking rate
	Additional feed is needed over an extended period
	Poor seasons are common
	Poor quality feed
Inexpensive	
Available feed not fully used e.g.	Low stocking rate
	Additional feed is needed over only a short period
	Good seasons are common
	High quality feed

### Inter-relation between feed and GHG measures

Feed intake and GHG emissions are related, and thus the recording of either one can provide information on the other that is useful in selection. For example, Robinson and Oddy [24] suggest that methane measurements could be used for predicting feed intake. The phenotypic relation between feed intake and GHG emission is strong when animals are forage-fed, but less strong when they are feedlot-fed. Only about half as much variation in GHG emissions is explained by feed intake under feedlot conditions [19]. This has ramifications for determining which measures are best for reducing livestock emissions [24, 25]. Measures that are independent of feed intake, such as measures of residual GHG, are likely to be more useful for intensive systems than for grazing systems. Moreover, reductions in total GHG emissions may be greater than those described here when production is entirely feedlot-based and when residual GHG emissions can be measured.

Other modelling of the example of production system in our study was conducted to simulate production entirely from pasture or entirely from grain [see Additional file 2]. The results showed that GHG emissions increased when production was from pasture and GHG emission costs were ignored, and they decreased when production was from grain. Although feed intake is known to be not as accurately predicted when feed intake is from grain [19], this also emphasises that the capacity to decrease GHG emissions depends on the way feed cost is modelled.

A comment is also warranted on two practical situations that can be encountered. Beef cattle selection is sometimes implemented without any consideration of the cost of feed. Our results suggest that this would increase total GHG emission for the production system by 6.2% per generation, or by ~ 12.4% in 10 years, and decrease net return for the production system by 1.7% per generation. Selection also sometimes occurs for animals that have only records on growth as against records for the range of traits that affect the breeding objective. Selection against growth in this circumstance would increase net returns by a small amount, but any advantage from this would be reduced when selection is not for a defined breeding objective because selection for a defined breeding objective takes all changes into account.

### Change in the relation between feed intake and GHG emission

In the example of production system in this study, selection reduced GHG emissions per unit of feed and the size of this reduction changed with the price of carbon (Figs. 4g and 6g). This shows that the relation between GHG emissions and feed intake is not constant and that it may change under selection when a carbon price is applied. Thus, the phenotypic association between feed intake and GHG emission may need to be adjusted periodically, in accordance with the carbon price used in the breeding objective, when the aim is to reduce GHG emissions.

### Other reductions in GHG emissions

The literature is not clear on how GHG emissions vary when stocking rates change [26, 27]. In the absence of genetic selection, Clarke et al. [26] showed that GHG emissions per unit of feed increased when stocking rate increased. Our results show that selection reduced GHG emissions per unit of feed when feed was expensive (Table 6), which might align with a situation where stocking rates are high.

Hristov et al. [28] have reviewed management options for reducing GHG emissions from livestock. Other methods for reducing GHG emissions in cattle include supplementation with red algae from seaweed, for which sizeable reductions in emissions have been achieved [29]. If societal pressures continue to focus on reduction in GHG emissions from beef cattle, all available technologies may be needed. Our results show that genetic selection will be an important part of any strategy to reduce GHG emissions, and this can be achieved using a low price of carbon when feed is otherwise expensive. When feed is inexpensive, greater growth will be favoured, leading to increased GHG emissions that may not be desirable.

## Conclusions

When the cost of feed in the breeding objective is high, multiple-trait selection can reduce total GHG emissions while increasing the economic performance of beef cattle using a low price for carbon. In the example of this article, total GHG emissions were reduced at a carbon price of less than $10/ton CO_2_-e. Both total GHG emissions and GHG emissions per unit of product were reduced. When the cost of feed in the breeding objective is low, selection can reduce total GHG emissions only if the price of carbon is high (about $80/ton CO_2_-e). Selection needs to be included in any strategy to reduce GHG emissions in beef cattle. When the cost of feed for the breeding objective is inexpensive, beef cattle selection that ignores emission costs will substantially increase GHG emissions.

## Additional files

**Additional file 1.** Production system calculations.

**Additional file 2.** Further modelling.

## References

[1] Hegarty RS, Goopy JP, Herd RM, McCorkell B (2007). Cattle selected for lower residual feed intake have reduced daily methane production. J Anim Sci.

[2] Herd RM, Arthur PF, Donoghue KA, Bird SH, Bird-Gardiner T, Hegarty RS (2014). Measures of methane production and their phenotypic relationships with dry matter intake, growth, and body composition traits in beef cattle. J Anim Sci.

[3] Gerber PJ, Steinfeld H, Henderson B, Mottet A, Opio C, Dijkman J (2013). Tackling climate change through livestock—A global assessment of emissions.

[4] Edenhofer O, Pichs-Madruga R, Sokona Y, et al. Technical summary. In: Edenhofer O, Pichs-Madruga R, Sokona Y, et al., editors. Climate change 2014: Mitigation of climate change. Contribution of working group III to the fifth assessment report of the intergovernmental panel on climate change. Cambridge: Cambridge University Press; 2014.

[5] Blaxter KL, Clapperton JL (1965). Prediction of the amount of methane produced by ruminants. Br J Nutr.

[6] Quinton C, Hely FS, Amer PR, Byrne TJ (2018). Prediction of effects of beef selection indexes on greenhouse gas emissions. Animal.

[7] Amer P, Fennessy P. Breeding for reduced greenhouse gas intensity of Australian livestock production. North Sydney: Final report to Meat & Livestock Australia; 2012.

[8] Wall E, Ludemann C, Jones H, Audsley E, Moran D, Roughsedge T, Amer P. The potential for reducing greenhouse gas emissions for sheep and cattle in the UK using genetic selection. Final report to DEFRA. Scottish Agricultural College; 2010.

[9] Barwick SA, Henzell AL, Walmsley BJ, Johnston DJ, Banks RG (2018). Methods and consequences of including feed intake and efficiency in genetic selection for multiple-trait merit. J Anim Sci.

[10] Graser HU, Tier B, Johnston DJ, Barwick SA (2005). Genetic evaluation for the beef industry in Australia. Aust J Exp Agric.

[11] Barwick SA, Henzell AL (2005). Development successes and issues for the future in deriving and applying selection indexes for beef breeding. Aust J Exp Agric.

[12] Schneeberger M, Barwick SA, Crow GH, Hammond K (1992). Economic indices using breeding values predicted by BLUP. J Anim Breed Genet.

[13] Freer M, Dove H, Nolan JV (2007). Nutrient requirements of domesticated ruminants.

[14] Alford AR, Hegarty RS, Parnell PF, Cacho OJ, Herd RM, Griffith GR (2006). The impact of breeding to reduce residual feed intake on enteric methane emissions from the Australian beef industry. Aust J Exp Agric.

[15] Arthur JPF, Herd RM (2008). Residual feed intake in beef cattle. R Bras Zootech.

[16] Waghorn GC, Hegarty RS (2011). Lowering ruminant methane emissions through improved feed conversion efficiency. Anim Feed Sci Technol.

[17] Herd RM, Velazco JI, Arthur PF, Hegarty RF (2016). Associations among methane emission traits measured in the feedlot and in respiration chambers in Angus cattle bred to vary in feed efficiency. J Anim Sci.

[18] Charmley E, Williams SRO, Moate PJ, Hegarty RS, Herd RM, Oddy VH (2015). A universal equation to predict methane production of forage-fed cattle in Australia. Anim Prod Sci.

[19] Johnson DE, Hill TM, Ward GM, Johnson KA, Branine ME, Carmean BR, Khalil MAK (1993). Ruminants and other animals. Atmospheric methane: Sources, sinks, and role in global change.

[20] Turner BW, Alcock DJ (2000). The dry sheep equivalent—redefining a ‘standard’. Asian-Aus J Anim Sci.

[21] Davies L, Mortensen K. Leasing land—calculating a rental. Prime Fact 338: NSW Department of Primary Industries; 2007.

[22] AusMeat. Handbook of Australian meat. 7th Ed. Sydney: AusMeat; 2006.

[23] de Haas Y, Windig JJ, Calus MPL, Dijkstra J, de Haan M, Bannink A (2011). Genetic parameters for predicted methane production and potential for reducing enteric emissions through genomic selection. J Dairy Sci.

[24] Robinson DL, Oddy VH (2016). Benefits of including methane measurements in selection strategies. J Anim Sci.

[25] Pickering NK, Oddy VH, Basarab J, Cammack K, Hayes B, Hegarty RS (2015). Animal board invited review: genetic possibilities to reduce enteric methane emissions from ruminants. Animal.

[26] Clarke AM, Brennan P, Crosson P (2013). Life-cycle assessment of the intensity of production on the greenhouse gas emissions and economics of grass based suckler beef production systems. J Agric Sci.

[27] Henry B, Eckard R (2009). Greenhouse gas emissions in livestock production systems. Trop Grassl.

[28] Hristov AN, Ott T, Tricarico J, Rotz A, Waghorn G, Adesogan A (2013). Mitigation of methane and nitrous oxide emissions from animal operations III. A review of animal management mitigation options. J Anim Sci.

[29] Kinley RD, de Nys R, Vucko MJ, Machado L, Tomkins NW (2016). The red macroalgae *Asparagopsis taxiformis* is a potent natural antimethanogenic that reduces methane production during in vitro fermentation with rumen fluid. Anim Prod Sci.

